# Autoantibody profiling to follow evolution of lupus syndromes

**DOI:** 10.1186/ar3927

**Published:** 2012-07-27

**Authors:** Nancy J Olsen, Quan-Zhen Li, Jiexia Quan, Ling Wang, Azza Mutwally, David R Karp

**Affiliations:** 1 Division of Rheumatology, Department of Medicine, Penn State MS Hershey Medical Center, 500 University Drive, Hershey PA 17033, USA; 2 Department of Immunology, University of Texas Southwestern Medical Center, 5323 Harry Hines Blvd, Dallas, TX 75390-9093, USA; 3 Rheumatic Diseases Division, Department of Medicine, University of Texas Southwestern Medical Center, 5323 Harry Hines Blvd, Dallas, TX 75390-8884, USA

## Abstract

**Introduction:**

Identification of patients who are in early stages of lupus is currently done through clinical evaluation and is not greatly facilitated by available diagnostic tests. Profiling for patient characteristics and antibody specificities that predict disease would enhance the ability of physicians to identify and treat early cases prior to onset of organ damaging illness.

**Methods:**

A group of 22 patients with 4 or fewer diagnostic criteria for lupus were studied for changes in clinical and autoantibody profiles after a mean follow up period of 2.4 years. An array with more than 80 autoantigens was used to profile immunoglobulin G (IgG) and immunoglobulin M (IgM) autoantibodies. Correlations with clinical disease progression were examined.

**Results:**

3 of the 22 patients (14%) added sufficient criteria during follow up to satisfy a diagnosis of systemic lupus erythematosus (SLE) or to acquire a diagnosis of SLE renal disease. Patients who progressed were all females and were younger than those who did not progress (*P*=0.00054). IgG but not IgM autoreactivity showed greater increases in the progressor group than in the non-progressor group (*P*=0.047). IgG specificities that were higher at baseline in progressors included proliferating cell nuclear antigen (PCNA), beta 2 microglobulin, C1q and hemocyanin (*P*<0.019). Progressors had significant increases in La/SSB and liver cytosol type 1 (LC1) IgG autoantibodies over the period of evaluation (*P*≤0.0072). A quantitative risk profile generated from baseline demographic and autoantibody variables yielded highly different scores for the progressor and non-progressor groups (*P*=1.38 × 10^-7^)

**Conclusions:**

In addition to demographic features, autoantibody profiles using an expanded array of specificities were correlated with the risk of progressive disease in patients with lupus. These findings suggest the feasibility of developing a simple diagnostic that could be applied by nonspecialists to screen for lupus and permit effective triage for specialty care.

## Introduction

The onset of systemic lupus erythematosus (SLE) may be an insidious process that can go unrecognized by the patient or by the primary care provider in the early phases. This is in part because the symptoms of SLE are heterogeneous and involve disparate organ systems and also due to the fact that early manifestations such as sun sensitivity, skin rash, chest discomfort and joint pains are relatively common complaints that are usually associated with non-SLE causes [1,2]. Conversely, the most serious organ involvement, acute kidney injury, may go completely unrecognized in the early stages. Another impediment to early detection is that patients with SLE usually have onset at relatively young ages when chronic organ-damaging illness is unexpected.

When SLE is suspected, testing for antinuclear antibodies (ANA) is usually carried out. While ANA positivity is associated with SLE in almost all cases, the high prevalence of ANA positivity in the general population, which approaches 25%, means that most individuals with ANAs do not in fact have SLE or a related autoimmune disease. So the interpretation of ANA positivity in the setting of vague symptoms is not straightforward, and the many positive results obtained in practice distract focus away from the few individuals who are in fact at high risk [3].

Improving the accuracy of early SLE diagnosis would be greatly aided by the availability of blood markers that have greater disease specificity in the early stages. One approach to accomplishing this objective is to expand profiling for autoantibody expression with the goal of identifying specificities that signal a high risk of future disease [4]. Since SLE is associated with more than 100 different autoantibodies [5] and many are present years prior to SLE diagnosis [6], it is reasonable to postulate that some of these might appear early and herald future disease.

A useful group of patients for study of early SLE are those individuals who have some features of SLE but who do not have a full complement of the defined criteria needed to establish the diagnosis with certainty. These individuals have been designated as having incomplete lupus, or ILE, and it is likely that a subset of these patients is in the early stages of SLE [7,8].

The present study was designed to determine whether clinical evolution of lupus-related autoimmunity can be correlated with expressed autoantibodies. An objective is to identify specificities that may be useful for distinguishing those individuals who are at high risk of disease progression. These studies made use of a slide-based autoantigen array for 80 specificities that has been described previously [9]. The findings suggest that development of a risk scale for lupus progression can be developed using demographic and autoantibody profile data.

## Materials and methods

### Study subjects

Serum samples were from the Dallas Regional Autoimmune Disease Registry (DRADR), which has been described previously [9,10]. Patients were recruited from at the University of Texas Southwestern Medical Center clinics at Parkland Hospital and the Aston Ambulatory Care Center. Additional patients were from a local community-based practice. Classification of clinical syndrome or disease was made at the time of registry enrollment. Patient examination, interview and medical record review were used to determine the presence of SLE criteria. Individuals with fewer than four criteria were designated as incomplete lupus (ILE). All individuals provided written informed consent for enrollment in the registry and the research carried out has been approved by the UT Southwestern Institutional Review Board.

Samples from 22 individuals in DRADR with four or fewer SLE criteria, and who had at least two samples drawn in the past 5 years, were utilized for this analysis (Table 1). The mean (standard error of the mean, SEM) age at the time of the baseline enrollment visit was 48.5 (2.4) years, with a range of 27 to 72 years, and the majority of samples (86%) were from females. The mean number of lupus criteria present at baseline was 2.1 (SEM, 0.2). One individual who was enrolled as ILE was found later to have four criteria, and thus had 4 criteria at the time of enrollment. No patients were on high-dose corticosteroids or cyclophosphamide during the period of study. There were no clinical criteria utilized to identify which individuals to resample; the second visit was determined only by availability. Patients had repeat samples drawn when they were encountered in our clinics or they were contacted to come to the research clinic for a repeat visit. The mean (SEM) time between blood draws was 2.4 (0.4) years and ranged from 0.5 to 6.5 years. Serum samples were processed and stored at -80°C in multiple aliquots.

**Table 1:** Characteristics of the study group

Characteristic	Value
Number of patients	22
Age (years)	48.5 ± 2.4*
Female gender (%)	86
African-American (%)	18
Hispanic/Latino (%)	18
ANA positive by ELISA (%)	82
ANA value (Elisa units)**	117.8 ± 19.0*
SLE criteria at Visit 1	2.1 ± 0.2*

*Values represent mean and standard error of the mean. **Positive values defined as > 20 Elisa units. ANA: antinuclear antibodies; SLE: systemic lupus erythematosus.

Each patient in DRADR has had measurement of ANA using an ELISA (Inova, San Diego CA), as described previously [11]. The positive ANA range in this assay is defined by the manufacturer as greater than 20 ELISA units (EU). The correlation between ELISA ANA measurement and the standard immunofluorescence assay (IFA) on Hep-2 cells has been examined previously in 15 DRADR patients with SLE. Scores for IFA positivity described as negative, positive and strongly positive had corresponding mean EU of 9.02 ± 2.9 (mean ± SEM), 31.0 ± 11.8 and 100 ± 22.4 and the correlation between the two measurements was statistically significant (*P *= 0.039). The average ANA was 117.8 (SEM, 19.0) EU, and four of the twenty-two individuals (18%) had ANA values less than 20 EU at the initial visit (Table 1). ANA measurements were repeated in 18 of the 22 subjects at least once and changes were not significant (*P *= 0.166). Notably, no individual developed or lost ANA positivity on repeat testing.

### Autoantigen array assays

All of the samples, including the first and second visits for each patient, were analyzed in one batch. Each serum sample was diluted as described previously and then applied to arrays in duplicate [9,11]. Autoantibodies were detected with Cy3-labeled anti-human IgG and Cy-5 labeled anti-human IgM on parallel arrays. Imaging and calculation of mean fluorescence intensity corresponding to the specificity of each autoantibody were carried out as described previously [11]. Values were expressed as normalized mean fluorescent intensities (MFI) using standard control antigens on each slide. Heat maps were generated using open source Cluster and Treeview programs (Eisen Software, Berkeley, CA, USA) [12]. The coefficient of variation for the slide-based array, based on repeat testing of patient and healthy control samples and considering all 80 specificities, is approximately 40%.

### Statistical analysis

Descriptive statistics were used to define variables, and data are shown as mean and SEM. Patient subgroups were compared by unpaired Student's *t*-test using Graph Pad Prism 5.0 (La Jolla, CA, USA). *P*-values < 0.05 were considered significant, except where otherwise indicated. Correlations for multiple comparisons were not carried out and the data analyses should be considered descriptive.

## Results

### Progressors and nonprogressors

Review of medical records and information collected at the return visit were utilized to determine whether the patients had accumulated additional criteria after the initial blood sampling [13,14]. Three of the twenty-two patients (14%) acquired additional lupus criteria over an average follow-up period of 3.8 ± 0.6 years (Table 2). Two of these were designated as ILE to SLE progressors and the third, who was enrolled as ILE but later recognized to have four criteria, acquired a new diagnosis of renal lupus. All three of these patients were designated as progressors (Prog). This rate of progression is similar to what has been reported by others [15]. The three progressors were female, with an age range of 27 to 33 years at the baseline visit, and their average age (30 years) was significantly lower than in the remaining nonprogressors (NProg), who had an average age of 51 years (range 35 to 72 years, *P *= 0.00054). Baseline ANA measured by ELISA tended to be higher in the Prog group (154 EU) than in the NProg group (112 EU), but this difference was not statistically significant (*P *= 0.46).

**Table 2:** Lupus features in study subjects at baseline and follow-up visits

Subject	Baseline SLE criteria*	Follow-up SLE criteria
1	ANA, heme, APL (RF)	
2	ANA, arthritis	
3	ANA (SSA)	
4	ANA, arthritis, Sm	
5	ANA	
6	ANA (SSA, SSB)	
7	ANA, photo, mucosal	
8	ANA, photo	
9	ANA, arthritis, dsDNA	
10	ANA, malar, serositis	
11	Arthritis, dsDNA	
12	ANA (RF)	
13	ANA	
14	ANA, arthritis	
15	ANA, heme	
16	ANA, photo	
17	ANA, photo, serositis	
18	ANA, discoid, heme	
19	ANA, photo (Scl70)	
20	ANA, photo, heme, dsDNA**	LN^† ^class V, mucosal, arthritis, serositis
21	ANA	discoid, photo, APL, heme
22	ANA	malar, discoid, photo, mucosal, arthritis, dsDNA

*Criteria are malar: malar rash; mucosal: mucosal ulcers; serositis: pleurisy, pericarditis; renal: renal disorder; heme: hematologic disorder; photo: photosensitivity. Immunologic disorder elements are listed separately (APL: antiphospholipid antibodies, dsDNA antibodies). Autoimmune features that are not part of systemic lupus erythematosus (SLE) criteria are listed in parentheses. **Patient was enrolled as ILE and subsequently found to have four SLE criteria. ^† ^LN: lupus nephritis.

### IgG autoantibodies

**Figure 1:**
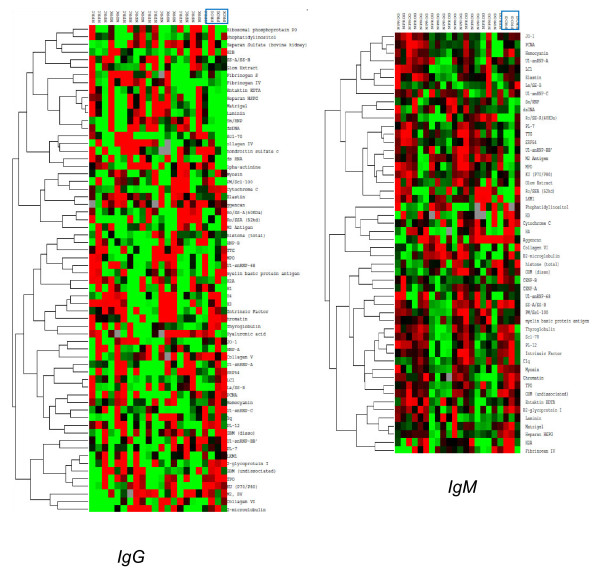
**Supervised clustering using Cluster/Treeview software was carried out with normalized signal intensities for baseline IgG (left) and IgM (right) autoantibodies in ILE patients**. Red represents values greater than the row mean and green represents values below that mean; gray corresponds to missing data. All Prog patients (progressors) are grouped together on the right side of each array and are indicated by rectangles.

The baseline IgG autoantigen array data for all individuals were examined using a supervised clustering algorithm (Figure 1). The Prog patients had downregulated levels of IgG antibodies to a group of structural proteins including entaktin, fibrinogens, heparin sulfate proteoglycan (HSPG) and laminin while other specificities related to nuclear components (chromatin, Ku), RNA (U1-snRNPs, Ro/SSA, La/SSB) and thyroid autoantigens (thyroid peroxidase, TPO, and thyroglobulin) were upregulated.

**Figure 2:**
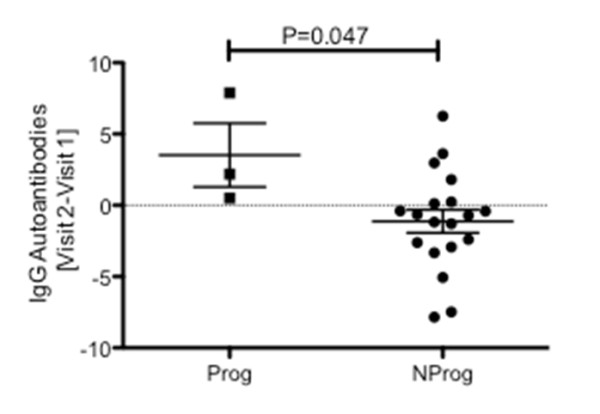
**Calculated change in total IgG autoreactivity between visit 1 and visit 2 in Prog (progressor) and NProg (nonprogressor ) subgroups**. Change was calculated for each autoantibody on the IgG array as visit 2 minus visit 1 values, and then these individual autoantibody change values were averaged for the whole array, yielding one overall result for each patient. Values > 0 correspond to increased IgG autoreactivity at visit 2. The two groups were compared using an unpaired Student's *t*-test.

Changes in IgG autoreactivity over time between the two visits were then calculated in two steps. First, differences in MFI value for each autoantibody were calculated as visit 2 minus visit 1 values. Then, the mean values for all IgG's on the array were computed for each individual subject. An increased value would reflect a higher burden of IgG class autoreactivity at the second visit. IgG autoreactivity was increased in all three of the ILE Prog individuals, while more than half of the NProg individuals with ILE had negative values, consistent with a decreased burden of IgG autoantibodies on follow-up (Figure 2). The difference between the two groups in change in overall IgG autoreactivity between visits was statistically significant (*P *= 0.047). This finding suggests that clinical progression is more likely to occur in patients who are accumulating additional IgG autoantibodies.

**Figure 3:**
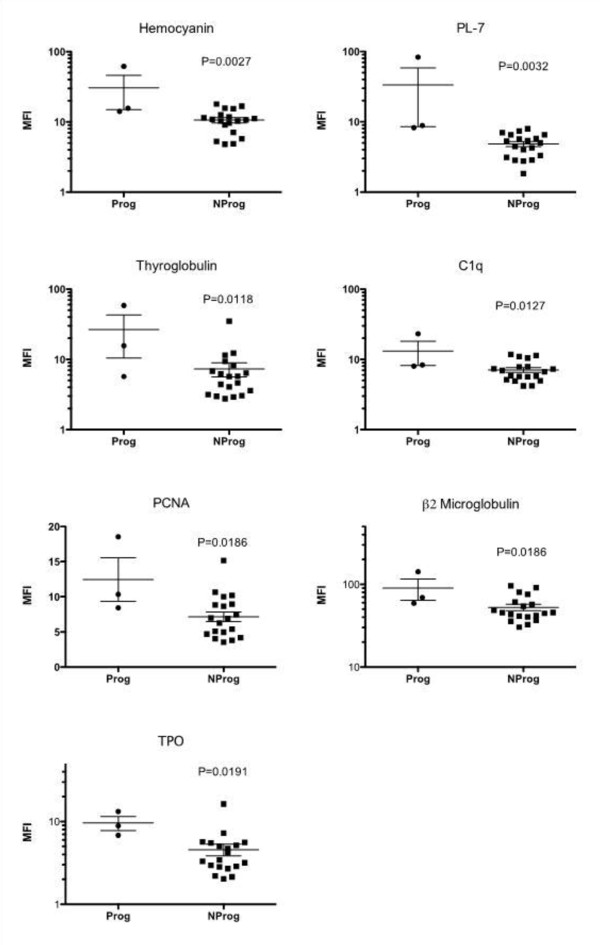
**Baseline levels of IgG autoantibodies in Prog (progressor) and NProg (nonprogressor) patient subsets**. Values represent mean fluorescence intensities (MFI). The two patient groups were compared using an unpaired Student's *t*-test. The seven autoantibodies shown represent those with significant differences (*P *< 0.05) between Prog and NProg subsets, which also had absolute expression values of at least 4 MFI.

Individual baseline IgG autoantibodies were then examined to identify those that were potentially useful in distinguishing subsequent ILE progression subgroups. Fifteen IgGs were significantly different (*P *< 0.05) in the Prog vs. NProg patient groups. Of these, seven had mean expression levels > 4.0 MFI units: hemocyanin, formiminotransferase cyclodeaminase or liver cytosol type 1 (LC1), thyroglobulin, C1q, proliferating cell nuclear antigen (PCNA), β2 microglobulin and TPO (Figure 3). Although each of these showed some overlap between Prog and NProg groups, it is apparent that patients with low expression levels for these IgGs uniformly did not progress to SLE or SLE/renal.

**Figure 4:**
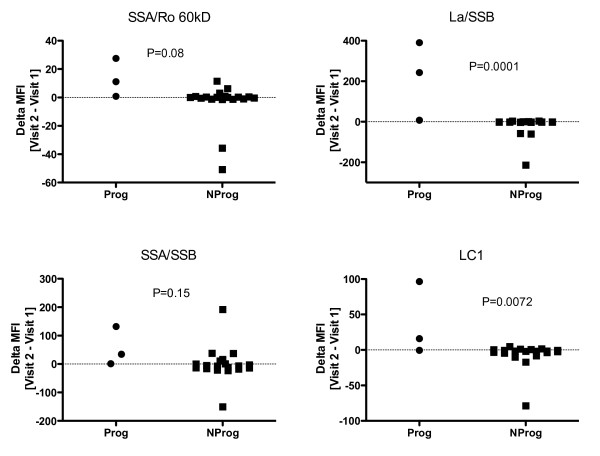
**Changes in mean fluorescence intensity (MFI) values between the two visits for four autoantibodies in the Prog (progressor) and NProg (nonprogressor) patient groups**. Two of these autoantibodies, La/SSB and LC-1, showed changes that were significantly related to progression status. The SSA/Ro 60 kD and SSA/SSB specificities showed marked changes in two of the three Prog patients. None of these four autoantibodies showed decreases in any of the Prog patients over the followup interval.

Examination of individual IgG autoantibodies in the two patient subsets revealed significant differences in values between the two visits at a level of *P *< 0.01 for six specificities: dsRNA, hemocyanin, La/SS-B, LC-1, PL-7 and TPO. Two of the three progressors showed increases in SS-A-related antigens, either Ro/SS-A 60kD or SS-A/SS-B and all three showed increases in La/SS-B. Notably, while some of the non-progressors showed decreases in these autoantibodies, none showed an increase in La/SS-B or LC-1 (Figure 4).

### IgM autoantibodies

**Figure 5:**
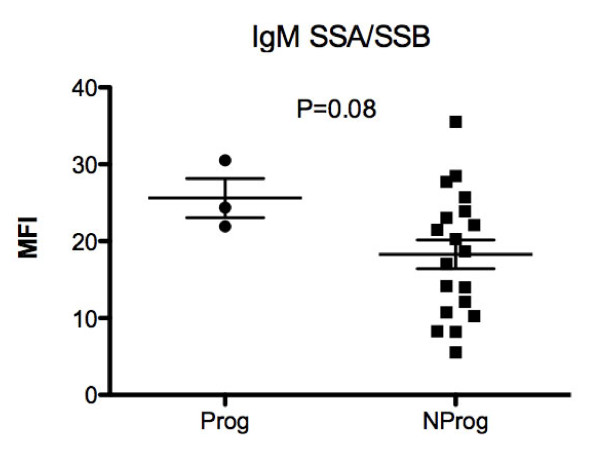
**Baseline mean fluorescence intensity (MFI) values for IgM autoantibodies directed against the SSA/SSB antigen**. No patients with initial IgM-SSA/SSB values less than 20 MFI showed progression of disease features during the follow-up period.

Absolute expression levels of IgM autoantibodies were generally lower than for IgG. The supervised clustering of the baseline IgM array showed generally lower levels of antibodies to RNA and nuclear antigens for the Prog patients while reactivity with some thyroid and structural proteins was increased (Figure 1, right panel). The highest absolute IgM values were observed for antibodies to the SSA/SSB autoantigen and a trend towards higher levels of IgM anti-SSA/SSB in Prog than in NProg patients was observed (*P *= 0.08) (Figure 5).

### Quantitative risk assessment

Several scoring systems for lupus risk were devised based on the data generated in this study. The first used demographic features alone as a linear three-point scale, assigning one point each for the presence of the following features: female gender, baseline ANA > 200 units, baseline age < 40 years. The Prog group of three patients had a mean score of 2.67 on this scale, which was significantly higher than the score of 1.15 in the NProg group (*P *= 6.3 × 10^-4^). Deriving a four-point scale by adding to this score one point for an increase in overall IgG autoreactivity on the array and adding zero points for decreased IgG autoreactivity, yielded scores of 3.67 and 1.47 in the two groups (*P *= 3.4 × 10^-4^). A five-point scale was then constructed incorporating values for the seven IgG autoantibodies that were significantly different at baseline in the Prog and NProg groups (Figure 2). To turn the specific IgG values into a single score, a value of 1 was assigned for expression levels above the overall mean for each autoantibody, and a value of 0 was assigned for levels below the mean, similar to the approach we have used previously in gene expression studies [16]. The values for all seven antibodies were totaled so that each patient had an autoantibody score in the range of 0 to 7. Adding this variable to create a five-point score yielded values of 10.00 in the Prog and 2.89 in the NProg groups (*P *= 1.38 × 10^-7^).

## Discussion

The search for readily-accessible blood biomarkers of disease risk is a major focus of biomedical research in many areas. The development over 50 years ago of a simple slide-based assay for serum ANAs is in fact an example of a paradigm-shifting biomarker. This single test was the key to the finding that patients with a wide array of clinical pathology in multiple organ systems, in fact shared a common autoimmune etiology [3]. Without the ANA blood test it would have taken far longer to define SLE and develop effective treatments. The current challenge is to find more specific indicators of high risk disease in early or even pre-clinical stages of SLE when interventions have significant disease-modifying potential [17,18]. We have used the designation of incomplete lupus (ILE) to identify individuals with less than 4 criteria, as a group that is at risk for progression of disease. One of the patients in the current study subsequently was recognized as having four criteria, although clinically, this individual was treated as ILE with hydroxychloroquine and subsequently progressed to develop renal involvement. This illustrates that although the ILE definition is somewhat arbitrary, it does have value in defining a population at risk for progressive illness.

A major tool employed in the current studies is the slide-based autoantigen array, which is in effect a scale-up of the classic slide-based ANA using a broad spectrum of autoantigens. Since many investigations have clearly documented that autoantibodies appear in the circulation prior to onset of clinically apparent SLE, it is reasonable to postulate that a subset of the autoantibodies on the array may be biomarkers predictive of risk [11]. The results presented here suggest that longitudinal evaluation of patients can provide useful insights into features that predict higher risk of progressive autoimmunity and support the hypothesis that the ILE patient category includes some individuals who are in evolving stages of disease [15]. The risk features include the relatively straightforward ones of female gender and age less than 40 years, which are also risk factors for development of ANA positivity itself [11]. Furthermore, having a high baseline ANA and an increase in IgG autoreactivity added to the risk score. The autoantigen arrays also point to a set of baseline IgG autoantibodies that further elevate the risk of progression to SLE. Validation of these observations and the proposed or alternative risk scores in a larger, prospectively collected sample will be required to determine whether these autoantibodies are in fact prognostic biomarkers.

Autoimmune responses modulate over time, and show patterns consistent with epitope spreading [19]. Antibodies to Ro/SSA are among the earliest to appear prior to SLE diagnosis, and responses to these epitopes show evidence of spread [20]. The present study showed that IgM and IgG antibodies Ro/SSA and La/SSB autoantigens may be related to disease progression. None of the patients with progression had decreases in IgG antibodies to La/SSB over the period of follow-up and conversely none of the nonprogressors showed increased levels of these autoantibodies. The La/SSB changes are of interest in view of animal model data demonstrating that the RNA recognition motif of La/SSB mediates epitope spreading by triggering B cells to diversify the expressed repertoire through a molecular mimicry mechanism [21]. Whether the early upregulation of La/SSB autoantibodies in humans has specificity for later development of SLE or other autoimmune disorders remains undetermined [22]. Since Ro/SSA and La/SSB autoantibodies are readily measured in the clinical laboratory, these could be clinically useful predictive tests as others have suggested [23]. On the other hand, the overall prevalence of Ro/SSA and La/SSB in SLE is not high enough to deliver sufficient sensitivity to be a useful screening tool. Furthermore, the thyroid autoantibodies might actually indicate that the autoimmune problem is primarily in that gland. Overall, the use of a multiplex set of antibodies to develop a risk score is likely to be a more robust approach.

A screening test for risk based on clinical, demographic and laboratory features would be potentially useful in the primary care setting to help with appropriate triage for specialty care. The high volume of ANA positive referrals to rheumatology clinics might be more efficiently managed if a risk assessment tool were available. A scale that could reliably identify patients at high risk for progression also would be potentially useful to identify candidates for preclinical treatment protocols designed to delay, ameliorate or prevent progression to SLE. Hydroxychloroquine is one candidate medication that might be used for this purpose, since other data suggest that use of this drug in early disease may delay the onset of SLE [24]. Other treatments such as Vitamin D, statins and peptide vaccination have been also discussed as potential preclinical interventions [4].

There are several significant limitations to the present study. These include the small sample size and retrospective design, which potentially introduces bias in patient selection. Furthermore, the relatively short period of evaluation may not have permitted capture of additional changes that might become manifest only with longer follow-up. Other findings indicate that at least some ILE or ANA-positive patients experience years of latency and accumulating autoimmunity prior to disease onset [25].

## Conclusions

Patients with autoimmune markers of lupus include a subset with evolving disease in whom SLE may become manifest. Female gender, age less than 40 years and high ANA levels are significant risk factors for progression of disease. Autoantibodies to Ro/SSA and La/SSB may be of additional use in detecting individuals at risk for SLE progression, and other autoantibodies, especially in the IgG class, also may have significant predictive value. Further longitudinal studies to determine the validity of the proposed risk profile will be of interest. Ultimately, application of these tools to the design of preclinical treatment strategies designed to ameliorate or prevent manifestations of SLE should be considered.

## Abbreviations

ANA: antinuclear antibodies; APL: antiphospholipid antibodies; DRADR: Dallas Regional Autoimmune Disease Registry; ELISA: enzyme-linked immunosorbent assay; EU: Elisa units; HSPG: heparin sulfate proteoglycan; IFA: immunofluorescence assay; ILE: incomplete lupus; LC1: liver cytosol type 1; MFI: mean fluorescence intensity; NProg: non-progressors; PCNA: proliferating cell nuclear antigen; PL-7: threonyl-tRNA synthetase; Prog: progressors; RF: rheumatoid factor; SLE: systemic lupus erythematosus; TPO: thyroid peroxidase.

## Competing interests

NJO has equity interest in ArthroChip LLC and research grants from Roche and Savient Pharmaceuticals. DRK has research grants from Human Genome Sciences and Centocor. The other authors have no competing interests.

## Authors' contributions

NJO and DRK designed the study and supervised collection and analysis of the samples. QZL and LW carried out and analyzed the array experiments. JQ performed other autoantibody measures. AM coordinated patient recruitment and collection of the clinical information. All authors have read and approved the manuscript for publication.

## References

[B1] Kurien BT Scofield RH Autoantibody determination in the diagnosis of systemic lupus erythematosus Scand J Immunol 2006 64 227 235 16918691 10.1111/j.1365-3083.2006.01819.x

[B2] Rahman A Isenberg DA Systemic lupus erythematosus N Engl J Med 2008 358 929 939 18305268 10.1056/NEJMra071297

[B3] Meroni P Schur P ANA screening: an old test with new recommendations Ann Rheum Dis 2010 69 1420 1422 20511607 10.1136/ard.2009.127100

[B4] Doria A Zen M Canova M Bettio S Bassi N Nalotto L Rampudda M Ghirardello A Iaccarino L SLE diagnosis and treatment: when early is early Autoimmun Rev 2010 10 55 60 20813207 10.1016/j.autrev.2010.08.014

[B5] Sherer Y Gorstein A Fritzler MJ Shoenfeld Y Autoantibody explosion in systemic lupus erythematosus: more than 100 different antibodies found in SLE patients Semin Arthritis Rheum 2004 34 501 537 15505768 10.1016/j.semarthrit.2004.07.002

[B6] Arbuckle M McClain M Rubertone M Scofield R Dennis G James J Harley J Development of autoantibodies before the clinical onset of systemic lupus erythematosus N Engl J Med 2003 349 1526 1533 14561795 10.1056/NEJMoa021933

[B7] Laustrup H Voss A Green A Junker P Occurrence of systemic lupus erythematosus in a Danish community: an 8-year prospective study Scand J Rheumatol 2009 38 128 132 19117248 10.1080/03009740802419073

[B8] Fabio G Carrabba M Hu C Floriani M Besana C Dramatic development of severe SLE in a patient with an incomplete disease Rheumatol Int 2005 25 543 547 15662528 10.1007/s00296-004-0550-1

[B9] Li Q Zhou J Wandstrat A Carr-Johnson F Branch V Karp D Mohan C Wakeland E Olsen N Protein array autoantibody profiles for insights into systemic lupus erythematosus and incomplete lupus syndromes Clin Exp Immunol 2007 147 60 70 17177964 10.1111/j.1365-2249.2006.03251.x PMC1810453

[B10] Wandstrat A Carr-Johnson F Branch V Gray H Fairhurst A Reimold A Karp D Wakeland E Olsen N Autoantibody profiling to identify individuals at risk for systemic lupus erythematosus J Autoimmun 2006 27 153 160 17052888 10.1016/j.jaut.2006.09.001

[B11] Li QZ Karp DR Quan J Branch VK Zhou J Lian Y Chong BF Wakeland EK Olsen NJ Risk factors for ANA positivity in healthy persons Arthritis Res Ther 2011 13 R38 21366908 10.1186/ar3271 PMC3132017

[B12] Open Source Clustering Software http://bonsai.ims.u-tokyo.ac.jp/~mdehoon/software/cluster/10.1093/bioinformatics/bth078 14871861

[B13] Tan E Cohen A Fries J Masi A McShane D Rothfield N Schaller J Talal N Winchester R The 1982 revised criteria for the classification of systemic lupus erythematosus Arthritis Rheum 1982 25 1271 1277 7138600 10.1002/art.1780251101

[B14] Hochberg MC Updating the American College of Rheumatology revised criteria for the classification of systemic lupus erythematosus Arthritis Rheum 1997 40 1725 9324032 10.1002/art.1780400928

[B15] Vilá LM Mayor AM Valentín AH García-Soberal M Vilá S Clinical outcome and predictors of disease evolution in patients with incomplete lupus erythematosus Lupus 2000 9 110 115 10787007 10.1191/096120300678828073

[B16] Maas K Chan S Parker J Slater A Moore J Olsen N Aune TM Cutting edge: molecular portrait of human autoimmune disease J Immunol 2002 169 5 9 12077221 10.4049/jimmunol.169.1.5

[B17] Pisetsky DS Antinuclear antibodies in healthy people: the tip of autoimmunity's iceberg? Arthritis Res Ther 2011 13 109 21554754 10.1186/ar3282 PMC3132028

[B18] Klareskog L Gregersen PK Huizinga TW Prevention of autoimmune rheumatic disease: state of the art and future perspectives Ann Rheum Dis 2010 69 2062 2066 21097657 10.1136/ard.2010.142109

[B19] Monneaux F Muller S Epitope spreading in systemic lupus erythematosus: identification of triggering peptide sequences Arthritis Rheum 2002 46 1430 1438 12115171 10.1002/art.10263

[B20] Heinlen LD McClain MT Ritterhouse LL Bruner BF Edgerton CC Keith MP James JA Harley JB 60 kD Ro and nRNP A frequently initiate human lupus autoimmunity PLoS One 2010 5 e9599 20224770 10.1371/journal.pone.0009599 PMC2835743

[B21] Routsias JG Kyriakidis N Latreille M Tzioufas AG RNA recognition motif (RRM) of La/SSB: the bridge for interparticle spreading of autoimmune response to U1-RNP Mol Med 2010 16 19 26 19838329 10.2119/molmed.2009.00106 PMC2762815

[B22] Defendenti C Atzeni F Spina MF Grosso S Cereda A Guercilena G Bollani S Saibeni S Puttini PS Clinical and laboratory aspects of Ro/SSA-52 autoantibodies Autoimmun Rev 2011 10 150 154 20854935 10.1016/j.autrev.2010.09.005

[B23] Sánchez-Guerrero J Lew RA Fossel AH Schur PH Utility of anti-Sm, anti-RNP, anti-Ro/SS-A, and anti-La/SS-B (extractable nuclear antigens) detected by enzyme-linked immunosorbent assay for the diagnosis of systemic lupus erythematosus Arthritis Rheum 1996 39 1055 1061 8651971 10.1002/art.1780390626

[B24] James JA Kim-Howard XR Bruner BF Jonsson MK McClain MT Arbuckle MR Walker C Dennis GJ Merrill JT Harley JB Hydroxychloroquine sulfate treatment is associated with later onset of systemic lupus erythematosus Lupus 2007 16 401 409 17664230 10.1177/0961203307078579

[B25] Heinlen LD McClain MT Merrill J Akbarali YW Edgerton CC Harley JB James JA Clinical criteria for systemic lupus erythematosus precede diagnosis, and associated autoantibodies are present before clinical symptoms Arthritis Rheum 2007 56 2344 2351 17599763 10.1002/art.22665

